# Fatty Acid Profile in Field-Collected Seaweed, Lipid Extraction Optimization, and Food Functional Properties

**DOI:** 10.3390/life15050710

**Published:** 2025-04-27

**Authors:** Nabeel Gnayem, Razan Unis, Rima Gnaim, Álvaro Israel, Jallal Gnaim, Alexander Golberg

**Affiliations:** 1 Department of Environmental Studies, Porter School of Environment and Earth Sciences, Tel Aviv University, Tel Aviv-Yafo 6997801, Israel; 2 The Triangle Regional Research and Development Center, Kfar Qari 3007500, Israel; 3 Israel Oceanographic and Limnological Research Institute, Haifa 3547133, Israel

**Keywords:** *Ulva comprressa*, polyunsaturated fatty acids, omega-3, omega-6, red seaweed, brown seaweed, functional food, lipid extraction

## Abstract

Macroalgae (seaweeds) represent a sustainable and alternative source of high-value fatty acids (FAs), including omega-3 (*n*-3) and omega-6 (*n*-6), which could help alleviate pressure on wild fish stocks and mitigate global overfishing. This study analyzed the FA composition of field-collected red (*Chondracanthus acicularis*, *Ballia callitriche*, *Gracilaria* sp., and *Gelidium coulteri*), brown (*Padina pavonica*, *Sargassum vulgare*, *Cystoseira myrica*, *Cystoseira* sp., *Dictyota dichotoma*, and *Stephanocystis mundane*), and green seaweeds (*Ulva compressa*). Additionally, lipid extraction was optimized using food-grade solvents, reaction temperatures, and the functional properties of selected green and brown seaweeds. The results showed that brown and green seaweeds contained higher total FA content than red seaweeds, with a favorable *n*-6/*n*-3 ratio below 10. The selected species’ functional properties (Water- and Oil-Holding Capacities, Swelling Capacity) met food-grade standards. Ethyl acetate at 60 °C was identified as the optimal food-grade solvent for lipid extraction. Functional properties varied significantly by species and processing conditions, with *Ulva compressa* exhibiting superior Water- and Oil-Holding Capacities and Swelling Capacity compared to *Cystoseira myrica*, highlighting its potential as a functional food ingredient. These findings support using seaweeds as a sustainable source for human nutrition.

## 1. Introduction

Seaweeds are considered a vital biomass source for third-generation biofuel production and excel in carbon assimilation during growth [1]. Cultivating or harvesting wild seaweeds could serve as an environmentally sustainable alternative to fishmeal and fish oil in the aquafeed industry [2]. Two aquaculture approaches—land-based and sea-based—are viable options for seaweed production, with land-based systems yielding higher protein and polyunsaturated fatty acid (PUFA) content while reducing heavy metal accumulation [3]. The nutritional value of *seaweeds* is heavily influenced by species selection [4].

The cultivation of seaweed and aquatic plants has expanded rapidly and is now practiced in approximately 50 countries [5]. In 2022, around 40 million tons were harvested for direct consumption or food processing, with traditional uses in Japan, the Republic of Korea, and China, as well as applications in fertilizers, pharmaceuticals, and cosmetics [5]. For centuries, humans have utilized seaweeds for food, hydrocolloid production (e.g., alginates and agar), medicine (e.g., treating iodine deficiency), and industrial uses such as paper, fertilizers, and animal feed [6].

Algae are rich in dietary fiber (non-digestible polysaccharides), vitamins, protein, minerals (absorbed and stored in their tissues), carotenoids, polyphenols, sulfolipids, and sulfated polysaccharides (e.g., fucoidan). Recently, marine seaweed exploitation has gained global attention, including in Portugal, particularly for pharmaceutical applications as a source of novel bioactive compounds [6]. Consequently, algae consumption is rising in Western countries.

Growing health awareness and recognition of omega-3 PUFAs’ nutritional benefits have driven demand for nutraceuticals [7]. Additionally, seaweeds are a sustainable source of polyphenols with significant antioxidant properties, highlighting their potential in health promotion and functional food applications [8]. Modern consumers increasingly seek nutrient-dense products with health benefits [9]. One strategy is incorporating algae into processed or fortified foods to enhance the nutritional value, bioactive compound content, and food quality, given their antioxidant and antimicrobial properties [10]. Some products, such as noodles and cookies, already include algae and *Cyanophyceae*, but further expansion of this trend is needed [11].

Algae composition varies (plasticity) depending on the species, harvest season, geographical location, and environmental conditions [9]. Lipid composition is particularly sensitive to environmental changes, leading to dynamic lipidome remodeling in response to biotic and abiotic stressors such as temperature fluctuations, salinity, light intensity, heavy metal exposure, and eutrophication [12]. Due to the high variability in *algal* lipid profiles, significant efforts have been made to develop efficient extraction and analytical methods for lipidome characterization [13].

Seaweeds are a rich source of high-value biochemicals, including lipids and PUFAs, essential for the food industry [1]. Omega-3 PUFAs, such as eicosapentaenoic acid (EPA) and docosahexaenoic acid (DHA), play crucial roles in human nutrition and metabolism [14]. These compounds are primarily derived from macro- and microalgae (e.g., diatoms) in marine ecosystems [2]. Although algae generally have a low lipid content, they contain high proportions of unsaturated fatty acids (UFAs) and PUFAs. Studies suggest that incorporating algae into diets may benefit health (e.g., managing diabetes or cancer), particularly when maintaining an omega-6/omega-3 ratio below 10 or 5 [7,15]. Elevated omega-6/omega-3 ratios are linked to cardiovascular, inflammatory, and autoimmune diseases, whereas higher omega-3 levels may mitigate these risks. Omega-3 sources include plants, fish, and algae, prompting the World Health Organization (WHO) to promote algae as a malnutrition intervention [16].

Optimizing lipid and fatty acid extraction from seaweeds is critical for assessing their potential as high-quality food and nutraceutical ingredients. Extraction methods must employ food-grade solvents to meet industry standards [17]. However, these solvents often yield lower lipid quantities and/or purity compared to conventional organic solvents (e.g., dichloromethane, methanol, chloroform), which, while efficient, pose toxicity risks and are non-compliant with food-grade regulations [18]. The choice of solvent, extraction method, and conditions influences fatty acid recovery, ratios, and profiles [19].

Functional food ingredients derived from seaweeds must align with industry standards, including the behavior of alga fine powder in water and oil to determine water- and Oil-Holding Capacities under varying blending conditions (e.g., temperature, duration) [20,21].

This work aimed to (1) evaluate the lipid content and fatty acid profile determined by gas chromatography–mass spectrometry (GC–MS) of a few green, red, and brown wild seaweeds collected over 3 years in three locations on the north coast of Israel on the Mediterranean Sea; (2) assess the efficiency of bio solvents such as ethanol and ethyl acetate in obtaining food-grade lipid ingredients from a few species of seaweed compared to traditional extraction methods and solvents; and (3) determine the functional properties of the dry powder of seaweeds, including the chewing capacity and Water- And Oil-Holding Capacity.

## 2. Materials and Methods

### 2.1. Field Collection of Seaweed Biomass

**Figure 1:**
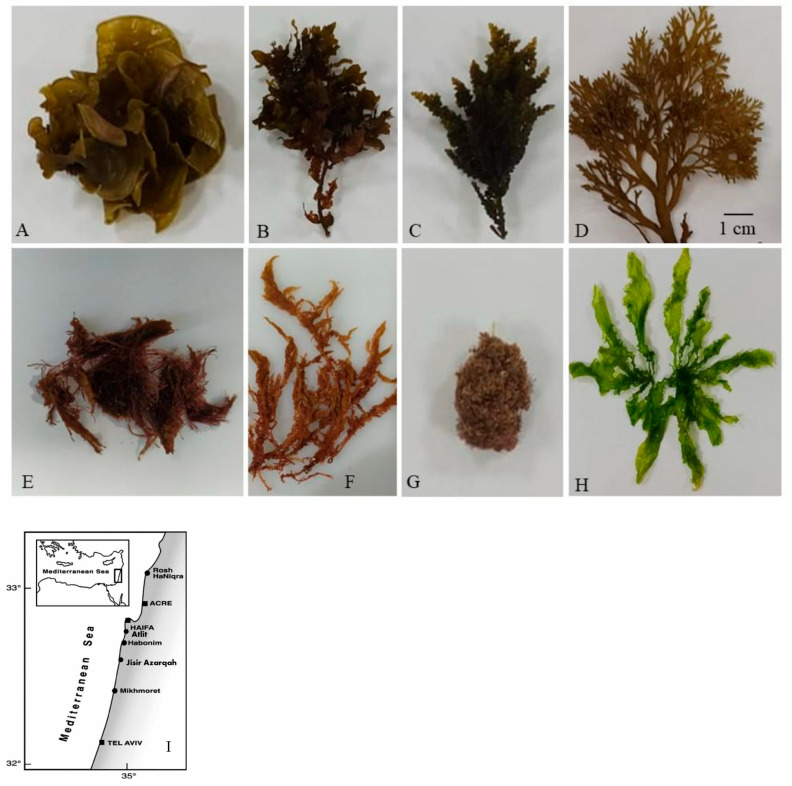
Pictures taken for the eight species of natural seaweeds collected at the three locations at the shores of the Mediterranean Sea, in northern Israel, on 27 May 2023. Letters represent the code of collected species: (**A**) *Padina pavonia*, (**B**) *Sargassum vulgare*, (**C**) *Cystoseira myrica*, (**D**) *Dictyota dichotoma*, (**E**) *Gelidium coulteri*, (**F**) *Gracilaria* sp. (**G**) *Ballia callitrichids*, and (**H**) *Ulva compressa*. Dimensions of species compared to the line of 1 cm. (**I**) Map of the north shores of Israel (following the map of Golani et al. 2007 [22]), Map of the Mediterranean coast of Israel showing the three study localities: Rosh Hanikra, Atlit, and Jisir Azarqah.

Overall, 13 seaweed samples—later identified as 8 species—were harvested on 27 May 2023 from three locations along the Israeli Mediterranean Sea: Rosh Hanikra (3 samples of 3 species), Atlit (3 samples of 3 species), and Jisr Az-Zarqa (8 samples of 8 species) (Table 1). During collection, seawater temperature, pH, and salinity were recorded, along with the exact coordinates (Table 2). The harvested seaweeds included three red seaweeds: *Ballia callitriche* (*B. callitriche*), *Gracilaria* sp., and *Gelidium coulteri* Harvey (*G. coulteri*); four brown seaweeds: *Padina pavonia* (Linnaeus) Thivy (*P. pavonica*), *Sargassum vulgare* C.Agardh (*S. vulgare*), *Cystoseira Myrica* (S.G.Gmelin) C.Agardh (*C. myrica*), *Dictyota dichotoma* (C.Agardh) Greville (*D. dichotoma*), and (*S. smundacea*); and one green seaweed: *Ulva compressa* Linnaeus (*U. compressa*). A complete list of species is provided in Table 1, with additional details in Supplementary Table S1 and morphological illustrations in Figure 1A–H. The sampling sites are shown on the map in Figure 1I.

After collection, the seaweeds were transported to the laboratory in plastic bags inside a cool box. The fresh biomass was rinsed with tap water to remove sand and debris. The fresh weight was recorded (n = 3), and the samples were then dried at 105 °C for 48 h until a constant weight was achieved. The dry weight was measured (n = 3), and the samples were stored at –80 °C until further analysis.

### 2.2. Morphological Identification of Natural Seaweeds

Macroscopic Examination: Specimens were rinsed with distilled water to remove epiphytes and debris. Morphological traits—including thallus shape, size, color, branching pattern, and holdfast structure—were examined and recorded using a stereomicroscope (Leica M205C, Wetzlar, Germany). Photographs were taken for comprehensive documentation.

Microscopic Examination: Thin sections of thalli were prepared using a razor blade and stained with 1% aqueous aniline blue to enhance cellular visualization. Microscopic features, such as cell arrangement, pyrenoid presence, and reproductive structures (e.g., sporangia and gametangia), were observed under a compound microscope (Nikon Eclipse E200, Amstelveen, The Netherlands) at 100× to 400× magnification.

Reproductive Structures: Special emphasis was placed on identifying reproductive structures, which are critical for taxonomic classification. The morphology and presence of spores, gametes, and other reproductive organs were meticulously documented.

### 2.3. Taxonomic Identification

Specimens were identified using dichotomous keys from standard taxonomic references, including Marine Algae of California [23] and Seaweeds of the World [24]. Morphological traits were compared with descriptions and illustrations from these sources.

Species Verification: taxonomic identification was cross-verified using the peer-reviewed literature, such as [25], which provides detailed morphological descriptions of Mediterranean seaweeds. Discrepancies were resolved through consultation with algal taxonomists and additional microscopic analysis.

### 2.4. Data Documentation

Morphological data and photographs were cataloged in a digital database for future reference and comparative studies. Quantitative traits—such as thallus length, branch diameter, and cell dimensions—were measured using ImageJ 1.54p software. Data were analyzed to assess intraspecific variation and differentiate closely related species. Cluster analysis (PAST 4.03 software) was employed to group specimens based on trait similarity. All morphological measurements and identifications were performed in triplicate to ensure accuracy. Expert validations were conducted by senior psychologists specializing in seaweed taxonomy from our research group (Israel Alvaro and Alexander Golberg). Cross-Identification: Fresh thalli were additionally cross-identified via visual morphology using AlgaeBase (https://www.algaebase.org/browse/taxonomy/) for species confirmation. Photographs of each collected species were archived as reference material [25,26].

### 2.5. Sample Preparation and Storage

Fresh biomass from each seaweed species was collected in triplicate (n = 3), with each sample stored separately as three independent replicates.

### 2.6. Dry Weight and Ash Content Determination

Fresh seaweed samples (n = 3) were weighed and dried at 105 °C for 48–72 h in pre-weighed crucibles using a conventional oven. The crucibles were then cooled in a desiccator, and the dry weight (DW) was recorded. DW was calculated using Equation (1). Subsequently, the dried biomass was combusted at 550 °C for 5 h in a muffle furnace. After combustion, the crucibles were removed, cooled in a desiccator, and reweighed to determine the ash content (AC) using Equation (2). All analyses were performed in triplicate.

DW and ash content (AC) were calculated using Equations (1) and (2), respectively.

DW (%) = 100% × (*m*_3_ − *m*_2_)/(*m*_1_ − *m*_2_) (1)

AC (%) = 100% × (*m*_4_ − *m*_2_)/(*m*_3_ − *m*_2_) (2)

where *m*_1_ is the mass of the fresh sample plus the crucible (mg), *m*_2_ is the mass of the crucible, *m*_3_ is the mass of the sample plus the crucible after drying at 105 °C, and *m*_4_ is the mass of the sample plus the crucible after combustion at 550 °C [27].

### 2.7. Elemental Analysis

The elemental composition (CHNS) of triplicate biomass samples (10 mg, dried at 105 °C) was determined using a Thermo Scientific Analyzer (Flash2000, Technion, Israel).

### 2.8. Extraction of Lipids

Lipid extracts were obtained following a slight modification of the standard method described by [28] and detailed by [29]. Briefly, dried biomass (100 mg) was placed in a glass centrifuge tube, followed by the addition of methanol (800 µL) and dichloromethane (500 µL) (Sigma, Hamburg, Germany, 98% purity). The mixture was vortexed, sonicated, and incubated in ice water on an orbital shaker (100 rpm, 150 min). After centrifugation (626× *g*, 10 min, 25 °C), the organic phase was separated. The solid residue was re-extracted with methanol (800 µL) and dichloromethane (500 µL) as described above. The combined supernatants yielded a clear pale green solution (2.5 mL).

Distilled water (1 mL) was added to the pooled organic solution, followed by centrifugation (626× *g*, 10 min, 25 °C) to separate the phases. The bottom organic phase was collected, microfiltered into a pre-weighed Eppendorf vial, and dried under an air stream (overnight in a fume hood at 25 °C). The final net weight was recorded.

### 2.9. Direct Methylation of FAs and Analysis by GC–MS

Dry seaweed biomass (25 mg), methanol (2 mL), dichloromethane (1 mL), concentrated H_2_SO_4_ (0.3 mL, 10% *v*/*v*), and *C*19:0 (20 µg, internal standard supplied by Sigma, Hamburg, Germany, 98% purity) were placed in a thick-walled glass pressure tube (5 mL) with a magnetic stirrer. The tightly sealed tube was heated at 90 °C overnight. After cooling to room temperature, the mixture was neutralized (pH 7) with NaOH solution (20% *w*/*v*) and transferred into a mixture of water and *n*-hexane (2:1 *v*/*v*, 5 mL). The upper organic phase was washed with double-distilled water (3 mL), filtered, and weighed following solvent evaporation [30], as described in the detailed methodology of Gnayem et al., 2025 [31].

### 2.10. Lipid Extraction Optimization

For lipid extraction optimization, the green seaweed *U. compressa*—collected from Jisr Azarqah on the Mediterranean coast of northern Israel on 27 May 2023—was selected due to its high lipid content. Triplicate samples of dry fine powder (1.5 g each) were mixed with 15 mL of five different organic solvents: methanol, ethanol, *n*-hexane, ethyl acetate, and dichloromethane (Sigma, Hamburg, Germany, 98% purity). The mixtures were vortexed in 22.5 mL vials and shaken at 150 rpm for 13 days at room temperature. The supernatant was filtered through Whatman paper into pre-weighed 18 mL plastic tubes and left to dry in a vented room for 13 days. The dry lipid weight was determined by subtracting the tube weight, and the total lipid percentage was calculated [17,18].

Temperature Optimization Test: Ethanol and ethyl acetate were selected for the temperature optimization test based on the aforementioned criteria, as they are suitable for food-grade extraction. A uniform batch of *U. compressa* seaweed (0.5 g per sample) was used for all extractions. A total of 24 samples were prepared, each diluted in 5 mL of solvent (ethanol or ethyl acetate, with 12 vials per solvent) at 30, 45, or 60 °C (4 vials per solvent–temperature combination). The mixtures were placed in sealed 22.5 mL vials with stirring magnets.

For each solvent, the 12 vials were divided into three temperature groups (n = 4 per group). After vortexing, the vials were placed in a water bath on a heating–stirring platform set to the respective temperatures. Extraction was conducted over 24 h. The supernatant was filtered through Whatman paper into pre-weighed 18 mL plastic tubes and left in a fume hood for 10 days to dry completely. To ensure uniform drying, the tubes were further incubated in an oven at 50 °C for 3 days. Finally, the tubes were weighed, and the total lipid percentage was calculated from the dried sample [19].

### 2.11. Functional Food Properties Determination

To assess the functional properties of *C. Myrica* and *U. compressa* seaweed powders (collected from Jisr Azarqah) for food-grade applications, three key tests were performed.

Water-Holding Capacity (WHC): Dry seaweed powder (1 g) from each species was mixed with 3 g of distilled water in pre-weighed 18 mL tubes (18 tubes total: 9 per seaweed, 3 per temperature). The samples were vortexed thoroughly and incubated for 1 h in a water bath at 25, 40, or 60 °C. After centrifugation at 3000× *g* for 25 min, the supernatant was removed via pipette, and the tubes were reweighed. WHC was calculated as grams of water retained per gram of dry sample [20,21].

Oil-Holding Capacity (OHC): The same experimental setup was used, replacing distilled water with corn oil (91.3 g per 100 mL). After incubation and centrifugation, OHC was determined as grams of oil retained per gram of dry sample [20,21].

#### Swelling Capacity (SC)

Dry seaweed powder (1 g) was hydrated with distilled water to a final volume of 10 mL in 18 mL tubes (3 tubes per seaweed, 6 total). The mixtures were vortexed and left at room temperature for 24 h. The swelling height was measured visually, and SC was expressed as cm^3^ per gram of dry sample [20,21].

### 2.12. Statistical Analysis

All measurements were performed in triplicate or quadruplicate (n = 3 or n = 4). Results are presented as mean ± standard deviation (SD). Statistical analysis was conducted using JMP IN software (version 5.0.1a, SAS Institute, Inc., Cary, NC, USA). One-way ANOVA followed by Tukey’s Honestly Significant Difference (HSD) test (*p* < 0.05) was applied to determine statistically significant differences between the means. The analysis revealed significant variations among some sample groups.

## 3. Results

### 3.1. Natural Field Seaweeds

The study sites were located along the northern shores of Israel, with sampling conducted on 27 May 2023 (Table 1). During collection, seawater salinity ranged between 3.45 and 3.51% (34.5–35.1 PPT), pH was 8.1–8.2, and temperature varied from 24.0 to 24.6 °C (Table 2), with fluctuations attributed to the sampling time.

Elemental analysis and ash content (Supplementary Table S1) revealed that the red seaweed *B. callitricha* had the highest ash content (83.1 ± 0.2%), followed by the brown seaweeds *C. myrica* (47.8 ± 0.38%), *P. pavonia* (46.9 ± 2.08%), and *D. dichotoma* (26.7 ± 0.15%). The green seaweed *U. compressa* exhibited the lowest ash content. The carbon content (%) was highest in *C. myrica* (37.9 ± 0.61), followed by *U. compressa* (31.9 ± 0.03), and lowest in *B. callitricha* (12.6 ± 0.40). The nitrogen content (%) was significantly higher in *C. myrica* (3.4 ± 0.11) compared to *B. callitricha* (0.4 ± 0.01).

### 3.2. Species Identification

Species were identified through morphological analysis, cross-referenced with Mediterranean seaweed databases [32,33]. Red, brown, and green seaweeds were selected for lipid and fatty acid profiling [26].

### 3.3. Fatty Acid Composition

Total Fatty Acids (TFAs): Eight seaweed species exhibited TFA concentrations ranging from 0.33 to 20.03 g kg^−1^ (Table 3, Table 4 and Table 5). The green seaweed *U. compressa* had the highest TFA, while the red seaweed *B. callitricha* had the lowest. Among brown seaweeds, *P. pavonia* and *C. myrica* contained 15.3 and 19.93 g kg^−1^ TFA, respectively.

Omega-3 and Omega-6 Fatty Acids: C18:3 *n*-3 (ALA) was highest in *P. pavonia* (21.9%) and *S. vulgare* (12.53%). C20:4 *n*-6 (ARA) was highest in *P. pavonia* (6.84%) and *S. vulgare* (8.35%). C16:0 (Palmitic acid) dominated TFA (45.07–97.15%), with the highest proportion in *U. compressa* (97.15%) (Table 4).

Fatty Acid Classes: Saturated fatty acids (SFAs) were highest in *U. copressa* (96.81%) and *B. callitricha* (97.66%) and lowest in *P. pavonia*. Polyunsaturated fatty acids (PUFAs) were highest in *P. pavonia* (45.91%) and *S. vulgare* (35.16%), driven by elevated *n*-3 (*P. pavonia*: 26.53%; *S. vulgare*: 12.96%) and *n*-6 (*P. pavonia*: 12.09%; *S. vulgare*: 10.99%) content. The n6/n3 ratio was highest in *B. callitricha* (5.02%), within acceptable limits for food-grade sources. Comparative analysis (Table 5) shows that brown seaweeds (*S. smundacea*, *D. dichotoma*) had significantly higher TFA (11.91 and 10.62 g kg^−1^) than red seaweeds (*G. coulteri*: 5.27; *G.* sp.: 3.57; *G.* sp.: 0.98 g kg^−1^). Regarding SFA, red seaweeds were richer in C16:0 (82.17–92.08%) compared to brown species (44.98–47.44%).

The PUFA content in brown seaweeds was higher than the C18:3 *n*-3 (*D. dichotoma*: 21.41%; *S. smundacea*: 16.6%), C20:4 *n*-6, and total *n*-3/*n*-6 content. All species exhibited n6/n3 ratios suitable for food applications.

### 3.4. Extraction Optimization

Solvent and Temperature Optimization for Lipid Extraction from *U. compressa*

**Figure 2:**
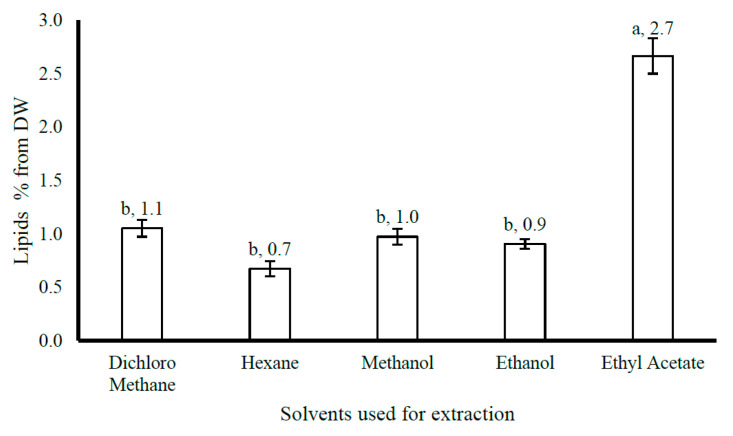
Lipid content in the selected seaweed *Ulva compressa* collected from Jisir Azarqah, located at the shore of the Mediterranean Sea, in north Israel on 27 May 2023 by 5 organic solvents under room temperature, expressed as (n = 3, error bars). Different letters near the values above the bars mean significant differences between the treatments.

**Figure 3:**
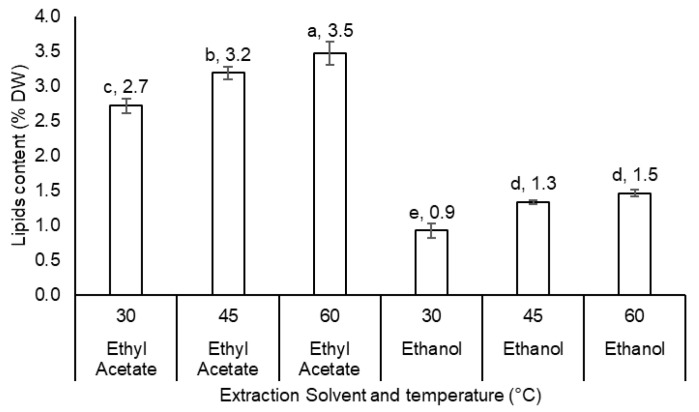
Lipid content in the selected seaweed *Ulva compressa* collected from Jisir Azarqah, located at the shore of the Mediterranean Sea, in north Israel on 27 May 2023 by 2 food grade solvents (ethyl acetate and ethanol) under 3 extraction temperature levels (30, 45, and 60 °C) expressed as (n = 4, error bars). Different letters near the values above the bars mean significant differences between the treatments.

Comparative analysis of solvents for lipid extraction from *U. compressa* (Figure 2) revealed that ethyl acetate yielded the highest lipid content at 2.7% (27 g kg^−1^), significantly outperforming dichloromethane (1.1%), *n*-hexane (0.7%), methanol (1.0%), and ethanol (0.9%). Given their food-grade suitability [17,18], ethyl acetate and ethanol were selected for further optimization. Temperature-dependent extraction (Figure 3) demonstrated that increasing the temperature from 30 °C to 60 °C significantly enhanced lipid yields: Ethanol: 0.92% (30 °C) → 1.33% (45 °C) → 1.46% (60 °C). Ethyl acetate: 2.72% (30 °C) → 3.19% (45 °C) → 3.47% (60 °C).

Notably, ethyl acetate consistently yielded significantly higher lipid concentrations than ethanol across all temperatures (*p* < 0.05). The results underscore the synergistic effect of solvent selection and elevated temperature on extraction efficiency.

### 3.5. Functional Food Properties

Functional Properties of Seaweed Powders for Food Applications. The functional properties of powders from two seaweed species—brown algae (*C. myrica*) and green algae (*U. compressa*)—collected from Jisir Azarqah on the Mediterranean coast of northern Israel (27 May 2023) were evaluated for their potential food industry applications.

**Figure 4:**
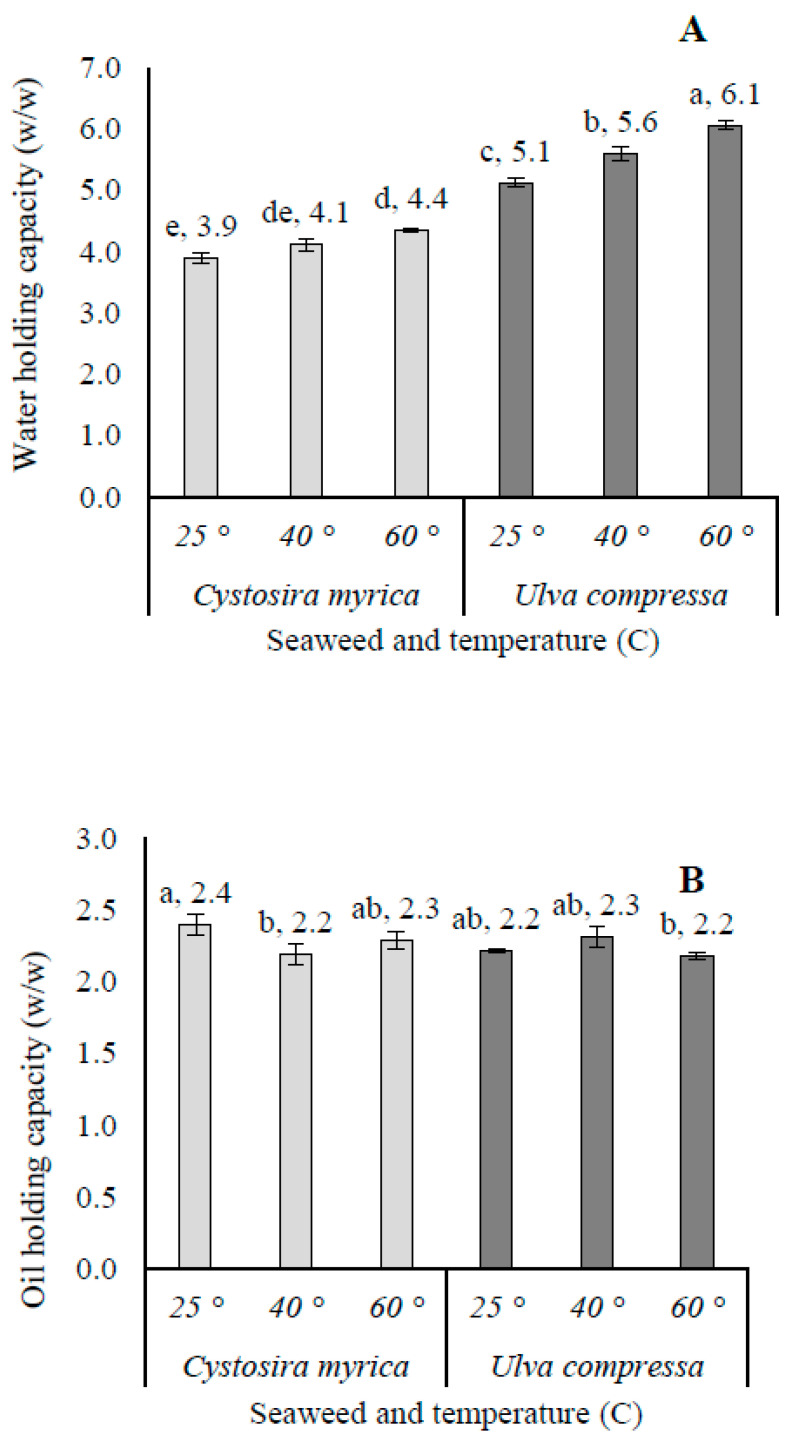
Functional properties for the food grade industry. (**A**) Water-Holding Capacity (*w*/*w*), (**B**) Oil-Holding Capacity (*w*/*w*) of powder from the selected two seaweed species (*C. myrica* and *U. compressa* collected from Jisir Azarqah, located at the shore of the Mediterranean Sea, in north Israel on 27 May 2023) as under 3 reaction temperature levels (25, 40, and 60 °C) expressed as (n = 3, error bars). Different letters near the values above the bars mean significant differences between the treatments.

Water-Holding Capacity (WHC) Analysis: The study revealed significant differences in WHC between species (*p* < 0.05): *U. compressa* demonstrated superior WHC (5.1–6.1%) across all tested temperatures (25, 40, and 60 °C). *C. myrica* showed lower WHC values (3.9–4.4%). Temperature dependence was observed for both species: *C. myrica* exhibited a clear temperature-dependent response, with WHC increasing progressively from 25 °C to 60 °C. *U. compressa* displayed more stable WHC values, with only a slight elevation at 40 °C. Statistical analysis (one-way ANOVA, Tukey’s HSD) confirmed significant differences (*p* < 0.05) between species at each temperature point, as indicated by letter groupings in Figure 4A. These findings suggest that *U. compressa* powder may offer superior functional properties for food applications requiring consistent water retention across processing temperatures.

Functional Properties of Seaweed Powders: Oil-Holding Capacity (OHC) analysis (Figure 4B). The study revealed distinct OHC patterns between species: *C. myrica*: showed a range of 2.3–2.4% across temperatures (25–60 °C), with a slight decrease at 60 °C. *U. compressa*: demonstrated consistent OHC (2.2–2.3%), peaking at 40 °C. Species effect: *U. compressa* exhibited significantly higher OHC than C. myrica at all temperatures (*p* < 0.05). Temperature effect: optimal OHC occurred at 40 °C for both species. *C. myrica* showed greater temperature sensitivity than *U. compressa.* Statistical significance: Letter annotations in Figure 4B indicate significant differences (*p* < 0.05) between treatments.

**Figure 5:**
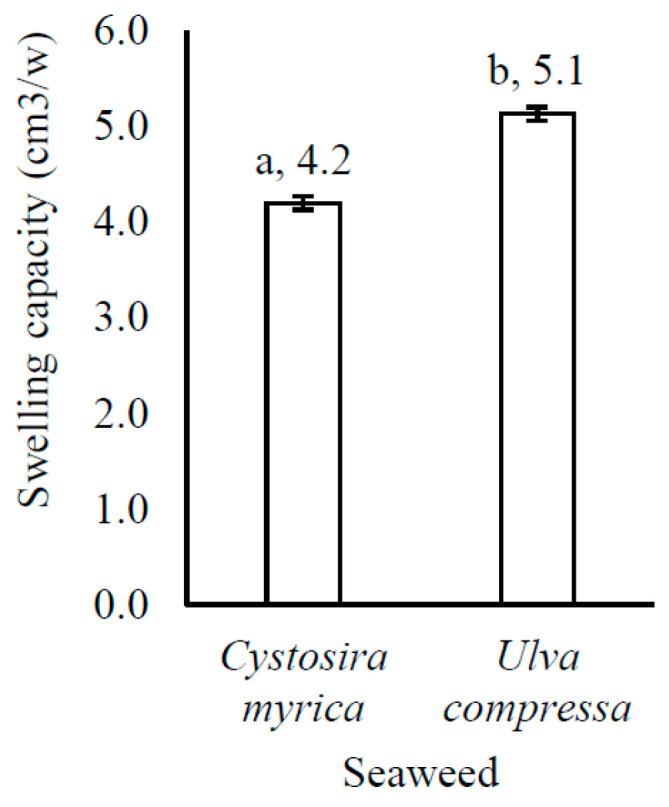
Swelling Capacity (cm^3^ w^−1^) of powder from two selected seaweed species (*Cystosira myrica* and *Ulva compressa* collected from Jisir Azarqah, located at the shore of the Mediterranean Sea, in northern Israel on 27 May 2023 as a functional property for the food-grade industry expressed as (n = 3, error bars). Different letters near the values above the bars mean significant differences between the treatments.

Swelling Capacity Analysis (SC) (Figure 5): The species exhibited markedly different swelling properties. *C. myrica* demonstrated superior Swelling Capacity (4.2 cm^3^ g^−1^), while *U. compressa* showed lower but consistent swelling (5.1 cm^3^ g^−1^).

## 4. Discussion

Analysis of fatty acid profiles across three seaweed phyla revealed significant diversity in the total fatty acid (TFA) content (3–20 g kg^−1^), with brown and green seaweeds exhibiting the highest concentrations. These findings align with [34] (*U. compressa*: 3–16.7 g kg^−1^), [35] (*U. lactuca*: 6–16 g kg^−1^), [36] (*Gracilaria* sp.: 42.5 ± 7.9 g kg^−1^), and [37,38] (*Sargassum* sp.: 25–33 g kg^−1^) for biorefinery applications.

The PUFA content differed markedly by phylum and location: Red/Brown seaweeds: Lower PUFA levels in Algerian coast specimens compared to Egyptian counterparts [39]. Green vs. Red seaweeds: *Ulva* sp. (13–37%) vs. *Gracilaria* sp. (3.23%) [40] and *Sargassum* sp. (13.92%) [41]. Nutritionally significant ratios and compounds show an *n*-6/*n*-3 ratio of 0.27–5.02 across species, suitable for food-grade applications [34]. Eicosapentaenoic Acid (EPA, C20:5 *n*-3) is prominent in brown seaweeds (*C. myrica*: 5.88%, *Cystoseira* sp.: 7.29%, and *D. dichotoma*: 2.78%) [42]. Red seaweeds (*Gracilaria* sp.) from Atlit and Rosh Hanikra showed minimal fatty acid content compared to sympatric brown seaweeds or green *U. compressa* (Table 4 and Table 5). Brown seaweeds consistently demonstrated superior fatty acid yields, highlighting their potential as nutraceutical sources [35,43,44].

### 4.1. Extraction Optimization

The lipid content of seaweed is a critical parameter for its potential applications in food, pharmaceuticals, and biofuels. The efficiency of lipid extraction depends on solvent selection and extraction conditions, particularly temperature. This study evaluates lipid extraction from *U. compressa*, a green seaweed from the Mediterranean Sea, using different solvents and temperatures. Lipid extraction from *U. compressa* was tested using five organic solvents at room temperature. The significant variations in lipid yields highlight the importance of solvent polarity and chemical properties. Polar solvents (e.g., ethanol and methanol) preferentially extract polar lipids, such as glycolipids and phospholipids, whereas non-polar solvents (e.g., *n*-hexane) are more effective for neutral lipids like triglycerides [45]. These differences likely arise from the varying solubility of lipid classes in each solvent. For example, chloroform–methanol mixtures are widely used due to their ability to solubilize diverse lipid classes [46]. These findings align with prior studies demonstrating that solvent choice critically influences lipid yield and composition in marine macroalgae [47,48]. The impact of temperature (30–60 °C) on lipid yield was assessed using two food-grade solvents: ethyl acetate and ethanol. Increasing the temperature significantly improved extraction efficiency, attributed to enhanced lipid solubility and better solvent penetration into the seaweed matrix [49]. Ethyl acetate, a moderately polar solvent, outperformed ethanol, likely due to its broader selectivity for both neutral and polar lipids. Although less efficient, ethanol is a greener and safer alternative for food and pharmaceutical applications [50]. This temperature-dependent trend agrees with studies on other seaweed species, where higher temperatures increased lipid recovery [51].

These findings emphasize the need to optimize solvent systems and extraction conditions to maximize lipid yields from *U. compressa*. Food-grade solvents like ethyl acetate and ethanol are particularly promising for nutraceuticals and functional foods. However, balancing extraction efficiency with environmental sustainability remains a challenge. Future research could explore advanced techniques (e.g., ultrasound-assisted or supercritical fluid extraction) to improve lipid recovery while reducing solvent use and energy consumption [52,53]. Further characterization of *U. compressa*’s lipid profile is also needed to evaluate its suitability for specific applications, such as omega-3 production or biodiesel feedstock.

### 4.2. Functional Food Properties

The functional properties of seaweed-derived powders, including Water-Holding Capacity (WHC), Oil-Holding Capacity (OHC), and Swelling Capacity (SC), are critical determinants of their suitability for food industry applications. These parameters significantly influence product texture, stability, and sensory characteristics. Figure 4 and Figure 5 present comparative data on these functional properties for powders derived from *C. myrica* and *U. compressa* collected from Jisr Azarqah, Israel, processed at different temperatures (25, 40, and 60 °C). The observed variations highlight the substantial impact of both species-specific characteristics and processing temperatures on these functional properties [54]. WHC and OHC, which reflect a material’s ability to retain water and oil, respectively, are largely determined by the polysaccharide composition, protein content, and fiber structure of seaweeds [55,56]. As demonstrated in Figure 4, *U. compressa* consistently showed superior WHC and OHC values compared to *C. myrica* across all temperature treatments. This enhanced performance can be attributed to the higher content of sulfated polysaccharides (particularly ulvan) in *Ulva* species, which demonstrate strong hydrophilic and lipophilic affinities [57]. The temperature-dependent increase in both WHC and OHC for both species suggests that thermal processing may enhance polysaccharide solubility and swelling, thereby improving their hydration and oil-binding properties [58].

The observed interspecies differences may be explained by their distinct biochemical compositions. *C. myrica*, a brown seaweed containing fucoidans and alginates, typically exhibits lower water and oil retention capacities compared to the sulfated polysaccharides predominant in *U. compressa* [59]. The statistically significant differences (denoted by letter groupings in Figure 4 and Figure 5) underscore the importance of temperature optimization for maximizing the functional performance of seaweed powders in food applications.

SC is a crucial parameter affecting texture, viscosity, and bulk density in food systems. Figure 5 reveals that *U. compressa* exhibited significantly greater SC than *C. myrica*, likely due to its high concentration of hydrophilic sulfated polysaccharides that readily absorb water and form gel matrices [60]. The swelling behavior of these polysaccharides is further influenced by molecular weight and degree of sulfation, with higher sulfation levels correlating with enhanced water absorption capacity [57].

These functional differences suggest distinct application profiles for each species, showing that *U. compressa* appears particularly suitable for applications requiring high viscosity and gel formation (e.g., soups, sauces, and dairy products), while *C. myrica* may be better adapted for products benefiting from lower swelling and firmer textures (e.g., baked goods or meat analogs) [57].

The superior WHC, OHC, and SC of *U. compressa* position it as an excellent candidate for use as a multifunctional ingredient (thickener, stabilizer, or emulsifier) in various food products. Conversely, *C. myrica* may find preferential use in low-moisture or low-fat formulations [53]. Temperature optimization emerges as a critical processing consideration, with moderate heating (40–60 °C) demonstrating beneficial effects on all measured functional properties, presumably through enhanced polysaccharide solubilization and swelling. However, additional research is warranted to evaluate potential trade-offs between functional property enhancement and the preservation of nutritional quality and sensory attributes at elevated processing temperatures [54]. Future studies should also investigate synergistic effects of combined temperature and mechanical processing, as well as the potential for targeted modification of polysaccharide structures to further optimize functional performance.

## 5. Conclusions

The present study addressed the potential of a few wild native Mediterranean Sea green, red, and brown seaweeds as a reservoir of bioactive compounds of fatty acids, focusing on PUFA, which is an essential component of human nutrition and has various health benefits. PUFA contains *n*-3 and *n*-6 FA, and an *n*-6/*n*-3 ratio is essential for the health implications, as the content in green and brown seaweeds is higher than that of red seaweed species, thus they are much relevant than red.

The lipid content of *U. compressa* is significantly influenced by the choice of solvent and extraction temperature. Food-grade solvents like ethyl acetate and ethanol and elevated temperatures offer a viable approach for efficient lipid extraction. These findings contribute to the growing body of knowledge on seaweed lipid extraction and highlight the potential of *U. compressa* as a sustainable source of lipids for various industries.

The functional properties of seaweed powders are highly dependent on the species and processing conditions. *U. compressa* demonstrated superior WHC, OHC, and SC compared to *C. myrica*, making it a valuable ingredient for the food industry. This study’s findings align with previous research on the functional properties of seaweed polysaccharides and provide valuable insights for the development of seaweed-based food products. Future research should focus on applying these powders in real food systems to evaluate their performance and consumer acceptance.

## Figures and Tables

**Table 1:** Seaweed types (green, red, or brown), species, and locations.

Seaweed Group	Species	Harvest Location		
		Rosh Hanikra	Atlit	Jisir Azarqah
Rhodophyta (Red)	*Gracilaria* sp.	+ *	+	+
	*Ballia callitriche*			+
	*Gelidium coulteri*			+
Ochrophyta (Brown)	*Padina pavonia*			+
	*Sargassum vulgare*			+
	*Cystoseira myrica*	+	+	+
	*Dictyota dichotoma*			+
Chlorophyta (Green)	*Ulva compressa*	+	+	+

* Means a sample of species is included and was collected from this location.

**Table 2:** Recorder GPS, PH, temperature, and salinity of seawater during seaweed harvesting in sites and times on 27 May 2023.

Site	GPS Location	Sea Water PH	Seawater Salinity %	Seawater Temperature (°C)	Harvesting Time
Rosh Hanikra	33°05′19″ E 35°07′02″ N	8.1	3.45	24.0	10:10
Atlit	34°56′25″ E 32°40′59″ N	8.1	3.46	24.6	13:00
Jisir Alzarqah	32°32′15″ E 34° 54′07″ N	8.1	3.51	24.5	16:30

**Table 3:** Fatty acid (FA) content and profile (% of total fatty acids, mean ± SD, n = 3, statistical analysis significance letter), saturated fatty acids (SFA, % of total fatty acids, mean ± SD, n = 3, statistical analysis significance letter), monounsaturated fatty acids (MUFA, % of total fatty acids, mean ± SD, n = 3, statistical analysis significance letter), polyunsaturated fatty acids (PUFA, % of total fatty acids, mean ± SD, n = 3, statistical analysis significance letter), omega-3 fatty acids (*n*-3, % of total fatty acids, mean ± SD, n = 3, statistical analysis significance letter), omega-6 fatty acids (*n*-6, % of total fatty acids, mean ± SD, n = 3, statistical analysis significance letter), omega-3/omega-6 ratio (*n*-6/*n*-3, % of total fatty acids, mean ± SD, n = 3, statistical analysis significance letter), in natural Brown seaweed samples species collected from three locations (Rosh Hanikra, Atlit, and Jisir Azarqah) on the shore of the Mediterranean Sea, in north Israel, on 27 May 2023. Fatty acid content was analyzed in triplicate (n = 3).

	Percentage of Fatty Acid (%) of Total Fatty Acids in Brown Seaweeds Collected from 3 Locations					
	JA ^11^	JA	RH	AT	JA	JA
Fatty Acid	*Padina pavoina* ^9^ (B) ^10^	*Sargassum vulgare* (B)	*Cystoseira myrica* (B)	*Cystoseira myrica* (B)	*Cystoseira myrica* (B)	*Dictyota dichotoma* (B)
C14:0	6.73 ± 0.40 b	8.36 ± 0.25 a	1.29 ± 0.05 c	1.35 ± 0.09 c	1.34 ± 0.06 c	0.78 ± 0.16 c
C16:4 *n*-4	0.42 ± 0.08 a	0.47 ± 0.10 a	0.13 ± 0.01 c	0.12 ± 0.01 c	0.13 ± 0.01 c	0.13 ± 0.01 c
C16:2 *n*-6	0.32 ± 0.03 a	0.13 ± 0.03 b	0.01 ± 0.00 c	0.02 ± 0.00 c	0.01 ± 0.00 c	0.01 ± 0.00 c
C16:1 *n*-5	0.06 ± 0.01 a	0.02 ± 0.01 a	0.00 ± 0.00 a	0.02 ± 0.00 a	0.00 ± 0.00 a	0.01 ± 0.00 a
C16:1 *n*-9	5.39 ± 0.28 b	8.84 ± 0.25 a	0.39 ± 0.05 c	0.37 ± 0.02 c	0.41 ± 0.05 c	0.27 ± 0.05 c
C16:0	45.07 ± 0.30 c	54.63 ± 2.12 b	86.82 ± 0.39 a	84.64 ± 0.10 a	85.72 ± 0.25 a	88.98 ± 0.26 a
C18:4 *n*-3	4.63 ± 0.82 a	0.42 ± 0.08 b	0.02 ± 0.00 c	0.03 ± 0.00 c	0.02 ± 0.00 c	0.02 ± 0.00 c
C18:2 *n*-6	4.94 ± 0.23 a	2.51 ± 0.36 b	0.38 ± 0.00 c	0.28 ± 0.01 c	0.31 ± 0.00 c	0.06 ± 0.01 d
C18:3 *n*-3	21.90 ± 0.76 a	12.53 ± 1.02 b	2.47 ± 0.18 c	2.44 ± 0.11 c	2.33 ± 0.15 c	0.76 ± 0.10 d
C18:1 *n*-7	1.42 ± 0.07 a	1.89 ± 0.01 a	0.26 ± 0.04 b	0.31 ± 0.04 b	0.28 ± 0.03 b	1.24 ± 0.07 a
C22:0	2.29 ± 0.09 a	1.85 ± 0.04 b	0.41 ± 0.04 c	0.42 ± 0.02 c	0.44 ± 0.03 c	2.37 ± 0.14 a
C20:4 *n*-6	6.84 ± 0.26 b	8.35 ± 0.30 a	1.91 ± 0.04 c	1.93 ± 0.11 c	1.85 ± 0.05 c	2.60 ± 0.17 c
C20:5 *n*-3 (EPA)	0.00 ± 0.00 c	0.00 ± 0.00 c	5.88 ± 0.44 a	6.05 ± 0.32 a	5.88 ± 0.37 a	2.78 ± 0.03 b
TFA ^1^	100.00 ± 0.00	100.00 ± 0.00	100.00 ± 0.00	100.00 ± 0.00	100.00 ± 0.00	100.00 ± 0.00
SFA ^2^	54.09 ± 1.59 c	64.84 ± 1.84 b	88.52 ± 0.00 a	86.41 ± 0.31 a	87.33 ± 0.36 a	92.14 ± 0.20 a
MUSFA ^3^	6.87 ± 0.33 b	10.75 ± 0.25 a	0.66 ± 0.05 d	0.70 ± 0.25 d	0.61 ± 0.05 d	1.51 ± 0.02 c
PUFA ^4^	45.91 ± 1.59 a	35.16 ± 1.84 b	11.48 ± 0.36 c	10.87 ± 0.31 c	10.62 ± 0.36 c	7.86 ± 0.20 d
*n*-3 ^5^	26.53 ± 1.58 a	12.96 ± 1.08 b	8.38 ± 0.36 c	8.52 ± 0.62 c	8.23 ± 0.36 c	3.56 ± 0.12 d
*n*-6 ^6^	12.09 ± 0.38 a	10.99 ± 0.67 b	2.31 ± 0.04 c	2.23 ± 0.10 c	2.26 ± 0.04 c	2.67 ± 0.17 c
n6/n3 ^7^	0.46 ± 0.02 b	0.85 ± 0.04 a	0.28 ± 0.02 c	0.26 ± 0.03 c	0.27 ± 0.02 c	0.75 ± 0.06 a
TFA ^8^ g kg^−1^	15.30 ± 1.12 b	8.28 ± 0.67 d	19.93 ± 1.49 a	19.49 ± 0.86 a	19.05 ± 1.49 a	13.94 ± 0.59 c

^1^ Total fatty acid. ^2^ Saturated fatty acid. ^3^ Monounsaturated fatty acids. ^4^ Polyunsaturated fatty acid. ^5^ Omega 3 fatty acids. ^6^ Omega 6 fatty acid. ^7^ Rate. ^8^ Total fatty acids content expressed as g kg^−1^ of dry weight. ^9^ Seaweed species tested. ^10^ B, Brown seaweed. ^11^ Location of sampling (RH) Rosh Hanikra, (AT) Atlit, and (JA) Jisir Azarqah. Different litters near values of the same line express significant differences using the one-way ANOVA Tukey HSD test (*p* < 0.05).

**Table 4:** Fatty acid (FA) content and profile (% of total fatty acids, mean ± SD, n = 3, statistical analysis significance letter), saturated fatty acids (SFA, % of total fatty acids, mean ± SD, n = 3, statistical analysis significance letter), monounsaturated fatty acids (MUFA, % of total fatty acids, mean ± SD, n = 3, statistical analysis significance letter), polyunsaturated fatty acids (PUFA, % of total fatty acids, mean ± SD, n = 3, statistical analysis significance letter), omega-3 fatty acids (*n*-3, % of total fatty acids, mean ± SD, n = 3, statistical analysis significance letter), omega-6 fatty acids (*n*-6, % of total fatty acids, mean ± SD, n = 3, statistical analysis significance letter), omega-3/omega-6 ratio (*n*-6/*n*-3, % of total fatty acids, mean ± SD, n = 3, statistical analysis significance letter), in natural Green seaweed samples species collected from three locations (Rosh Hanikra, Atlit, and Jisir Azarqah) on the shore of the Mediterranean Sea, in north Israel, on 27 May 2023. Fatty acid content was analyzed in triplicate (n = 3).

	Percentage of Fatty Acid (%) of Total Fatty Acids in Green Seaweed Collected from 3 Locations		
	RH ^11^	AT	JA
Fatty Acid	*Ulva compressa* ^9^ (G ^10^)	*Ulva compressa* (G)	*Ulva compressa* (G)
C14:0	0.13 ± 0.00 a	0.10 ± 0.00 a	0.12 ± 0.00 a
C16:4 *n*-4	0.08 ± 0.00 a	0.08 ± 0.00 a	0.06 ± 0.00 a
C16:2 *n*-6	0.23 ± 0.03 a	0.21 ± 0.02 a	0.22 ± 0.01 a
C16:1 *n*-5	0.01 ± 0.00 a	0.01 ± 0.00 a	0.01 ± 0.00 a
C16:1 *n*-9	0.26 ± 0.05 a	0.25 ± 0.08 a	0.22 ± 0.07 a
C16:0	96.18 ± 0.10 a	95.12 ± 0.14 a	97.15 ± 0.12 a
C18:4 *n*-3	0.11 ± 0.01 a	0.14 ± 0.01 a	0.13 ± 0.01 a
C18:2 *n*-6	0.04 ± 0.01 a	0.02 ± 0.01 a	0.03 ± 0.01 a
C18:3 *n*-3	0.25 ± 0.02 a	0.22 ± 0.02 a	0.23 ± 0.02 a
C18:1 *n*-7	1.50 ± 0.03 a	1.60 ± 0.02 a	1.40 ± 0.04 a
C22:0	0.30 ± 0.05 a	0.20 ± 0.05 a	0.40 ± 0.05 a
C20:4 *n*-6	0.02 ± 0.01 a	0.02 ± 0.01 a	0.03 ± 0.01 a
C20:5 *n*-3 (EPA)	0.01 ± 0.00 a	0.01 ± 0.00 a	0.01 ± 0.00 a
TFA ^1^	100.00 ± 0.00	100.00 ± 0.00	100.00 ± 0.00
SFA ^2^	97.66 ± 0.12 a	95.42 ± 0.12 a	97.66 ± 0.12 a
MUSFA ^3^	1.77 ± 0.11 a	1.86 ± 0.11 a	1.64 ± 0.11 a
PUFA ^4^	0.74 ± 0.12 a	0.70 ± 0.12 a	2.34 ± 0.12 a
*n*-3 ^5^	0.37 ± 0.04 a	0.37 ± 0.04 a	0.37 ± 0.04 a
*n*-6 ^6^	0.29 ± 0.02 a	0.25 ± 0.02 a	0.26 ± 0.02 a
n6/n3 ^7^	0.78 ± 0.10 a	0.68 ± 0.10 a	0.71 ± 0.10 a
TFA ^8^ g kg^−1^	19.53 ± 0.52 a	19.81 ± 0.61 a	20.03 ± 0.84 a

^1^ Total fatty acid. ^2^ Saturated fatty acid. ^3^ Monounsaturated fatty acids. ^4^ Polyunsaturated fatty acid. ^5^ Omega 3 fatty acids. ^6^ Omega 6 fatty acid. ^7^ Rate. ^8^ Total fatty acids content expressed as g kg^−1^ of dry weight. ^9^ Seaweed species tested. ^10^ G, Green seaweed. ^11^ Location of sampling (RH) Rosh Hanikra, (AT) Atlit, and (JA) Jisir Azarqah. Different litters near values of the same line express significant differences using the one-way ANOVA Tukey HSD test (*p* < 0.05).

**Table 5:** Fatty acid (FA) content and profile (% of total fatty acids, mean ± SD, n = 3, statistical analysis significance letter), saturated fatty acids (SFA, % of total fatty acids, mean ± SD, n = 3, statistical analysis significance letter), monounsaturated fatty acids (MUFA, % of total fatty acids, mean ± SD, n = 3, statistical analysis significance letter), polyunsaturated fatty acids (PUFA, % of total fatty acids, mean ± SD, n = 3, statistical analysis significance letter), omega-3 fatty acids (*n*-3, % of total fatty acids, mean ± SD, n = 3, statistical analysis significance letter), omega-6 fatty acids (*n*-6, % of total fatty acids, mean ± SD, n = 3, statistical analysis significance letter), omega-3/omega-6 ratio (*n*-6/*n*-3, % of total fatty acids, mean ± SD, n = 3, statistical analysis significance letter), in natural red seaweed samples species collected from three locations (Rosh Hanikra, Atlit, and Jisir Azarqah) on the shore of the Mediterranean Sea, in north Israel, on 27 May 2023. Fatty acid content was analyzed in triplicate (n = 3).

	Percentage of Fatty Acid (%) of Total Fatty Acids in Red Seaweeds Collected from 3 Locations				
	JA ^11^	AT	RH	AT	JA
Fatty Acid	*Ballia callitricha* ^9^ (R)	*Gelidium coulteri* (R)	*Gracilaria* sp. (R) ^10^	*Gracilaria* sp. (R)	*Gracilaria* sp. (R)
C14:0	0.54 ± 0.11 c	0.78 ± 0.09 c	2.00 ± 0.39 b	4.03 ± 0.10 a	3.41 ± 0.21 a
C16:4 *n*-4	0.29 ± 0.03 b	0.34 ± 0.04 b	0.05 ± 0.06 c	0.13 ± 0.01 c	0.46 ± 0.04 a
C16:2 *n*-6	0.67 ± 0.09 a	0.04 ± 0.03 c	0.19 ± 0.11 b	0.01 ± 0.00 c	0.11 ± 0.01 b
C16:1 *n*-5	0.20 ± 0.02 a	0.04 ± 0.03 b	0.01 ± 0.01 b	0.01 ± 0.00 b	0.01 ± 0.01 b
C16:1 *n*-9	0.20 ± 0.02 c	4.75 ± 0.04 b	0.32 ± 0.21 c	0.27 ± 0.05 c	6.91 ± 0.52 a
C16:0	82.21 ± 0.15 b	82.17 ± 0.10 b	92.08 ± 0.94 a	88.98 ± 0.26 a	69.09 ± 1.04 c
C18:4 *n*-3	0.14 ± 0.02 a	0.03 ± 0.02 b	0.04 ± 0.02 b	0.02 ± 0.00 b	0.07 ± 0.01 b
C18:2 *n*-6	0.77 ± 0.06 a	0.11 ± 0.08 b	0.05 ± 0.01 b	0.06 ± 0.01 b	0.15 ± 0.12 b
C18:3 *n*-3	0.13 ± 0.01 d	0.65 ± 0.04 c	1.42 ± 0.09 b	0.76 ± 0.10 c	4.65 ± 0.21 a
C18:1 *n*-7	0.14 ± 0.02 d	4.96 ± 0.18 b	1.67 ± 0.37 c	1.24 ± 0.07 c	11.08 ± 0.50 a
C22:0	14.06 ± 0.03 a	1.68 ± 0.11 b	2.07 ± 0.17 b	2.37 ± 0.14 b	2.31 ± 0.07 b
C20:4 *n*-6	0.53 ± 0.03 b	1.27 ± 0.18 b	0.02 ± 0.01 c	2.60 ± 0.17 a	1.68 ± 0.04 b
C20:5 *n*-3 (EPA)	0.12 ± 0.01 c	3.18 ± 0.09 a	0.09 ± 0.02 c	2.78 ± 0.03 b	0.07 ± 0.01 c
TFA ^1^	100.00 ± 0.00	100.00 ± 0.00	100.00 ± 0.00	100.00 ± 0.00	100.00 ± 0.00
SFA ^2^	96.81 ± 0.09 a	84.63 ± 0.18 b	96.15 ± 0.53 a	95.38 ± 0.29 a	74.81 ± 1.06 c
MUSFA ^3^	0.55 ± 0.03 d	9.75 ± 0.12 b	2.00 ± 0.56 c	1.51 ± 0.02 c	18.00 ± 1.01 a
PUFA ^4^	3.19 ± 0.09 d	15.37 ± 0.18 b	3.85 ± 0.53 d	7.86 ± 0.20 c	25.19 ± 1.06 a
*n*-3 ^5^	0.39 ± 0.01 d	3.86 ± 0.03 b	1.54 ± 0.09 c	3.56 ± 0.12 b	4.79 ± 0.22 a
*n*-6 ^6^	1.96 ± 0.09 b	1.42 ± 0.09 b	0.26 ± 0.12 c	2.67 ± 0.17 a	1.94 ± 0.15 b
n6/n3 ^7^	5.02 ± 0.28 a	0.37 ± 0.02 b	0.17 ± 0.08 c	0.75 ± 0.06 b	0.40 ± 0.03 b
TFA ^8^ g kg^−1^	0.33 ± 0.02 c	5.27 ± 0.14 a	3.57 ± 0.14 b	3.48 ± 0.06 b	3.32 ± 0.15 b

^1^ Total fatty acid. ^2^ Saturated fatty acid. ^3^ Monounsaturated fatty acids. ^4^ Polyunsaturated fatty acid. ^5^ Omega 3 fatty acids. ^6^ Omega 6 fatty acids. ^7^ Rate. ^8^ Total fatty acids content expressed as g kg^−1^ of dry weight, ^9^ Seaweed species tested. ^10^ R = Red seaweed. ^11^ Location of sampling (RH) Rosh Hanikra, (AT) Atlit, and (JA) Jisir Azarqah. Different litters near values of the same line express significant differences using the one-way ANOVA Tukey HSD test (*p* < 0.05).

## Data Availability

The original contributions presented in this study are included in the article/supplementary material. Further inquiries can be directed to the corresponding author.

## Supplementary Materials

The following supporting information can be downloaded at https://www.mdpi.com/article/10.3390/life15050710/s1: Table S1: The elemental content (%) in dry weight (DW) (N%, C%, S%, H% mean ± SD, n = 3), elemental rate (N/C mean ± SD, n = 3), and Ash content (%) in 8 natural seaweed spices (13 samples) from three locations in north Israel collected on 26 May 2023.
