# Supplementary material for: Fatty Acid Profile in Field-Collected Seaweed, Lipid Extraction Optimization, and Food Functional Properties
Source: Life (Basel). 2025 Apr 27;15(5):710. doi: 10.3390/life15050710 (PMC12112965; doi:10.3390/life15050710)
Supplement: Supplementary file 1 [file life-15-00710-s001.zip › life-3556285-supplementary.pdf]

**Table S1:** The elemental content (%) in dry weight (DW) (N%, C%, S%, H% mean ± SD, *n* = 3), elemental rate (N/C mean ± SD, *n* = 3), and Ash content (%) in eight natural seaweed spices (13 samples) from three locations on north Israel collected on 26.5.2023.

| **Seaweed Species** | **Location** | **Element content (%)** | | | | **Elemental Ratio** | **Ash content %** |
|---|---|---|---|---|---|---|---|
| | | **N %** | **C %** | **S %** | **H %** | **C/N *** | |
| ***Gracilaria* sp. (R)** *** | RH**** | 0.4 ± 0.01 f | 12.6 ± 0.40 f | 0.8 ± 0.03 e | 0.1 ± 0.14 h | 28.9 ± 0.45 a | 54.1 ± 0.23 a |
| ***Gracilaria* sp. (R)** | AT | 0.4 ± 0.02 f | 12.5 ± 0.42 f | 0.9 ± 0.03 e | 0.1 ± 0.15 h | 29.8 ± 0.47 a | 56.0 ± 0.22 a |
| ***Gracilaria* sp. (R)** | JA | 0.4 ± 0.01 f | 12.7 ± 0.43 f | 0.7 ± 0.03 e | 0.1 ± 0.17 h | 29.7 ± 0.44 a | 55.1 ± 0.21 a |
| ***Ballia callitricha* (R)** | JA | 0.5 ± 0.01 f | 12.9 ± 0.45 f | 0.9 ± 0.06 e | 0.2 ± 0.16 h | 29.9 ± 0.45 a | 56.1 ± 0.24 a |
| ***Gelidium coulteri* (R)** | JA | 1.1 ± 0.08 e | 19.8 ± 0.95 e | 3.1 ± 0.20 cd | 1.6 ± 0.13 f | 18.2 ± 0.80 c | 40.2 ± 0.48 d |
| ***Padina pavonia* (B)** | JA | 1.5 ± 0.10 cd** | 23.7 ± 1.17 d | 3.2 ± 0.23 c | 2.0 ± 0.06 e | 15.9 ± 0.37 de | 45.5 ± 0.22 bc |
| ***Sargassum vulgare* (B)** | JA | 1.3 ± 0.05 de | 26.8 ± 0.41 c | 3.8 ± 0.17 b | 1.4 ± 0.08 fg | 20.6 ± 0.84 b | 39.8 ± 1.16 d |
| ***Cystoseira myrica* (B*)*** | RH | 2.0 ± 0.05 b | 25.7 ± 0.40 cd | 4.1 ± 0.08 b | 3.6 ± 0.05 c | 12.7 ± 0.52 fg | 47.8 ± 0.38 b |
| ***Cystoseira myrica* (B)** | AT | 3.4 ± 0.11 a | 37.9 ± 0.61 a | 5.7 ± 0.07 a | 1.3 ± 0.04 fg | 11.2 ± 0.19 g | 29.0 ± 0.49 f |
| ***Cystoseira myrica* (B)** | JA | 3.4 ± 0.11 a | 38.9 ± 0.71 a | 5.9 ± 0.07 a | 1.5 ± 0.04 fg | 12.2 ± 0.19 g | 28.0 ± 0.45 f |
| ***Dictyota dichotoma* (B)** | JA | 1.7 ± 0.02 c | 24.9 ± 0.28 cd | 4.0 ± 0.07 b | 4.3 ± 0.07 b | 15.0 ± 0.19 de | 46.9 ± 2.08 bc |
| ***Ulva compressa* (G)** | RH | 1.7 ± 0.00 c | 33.8 ± 0.05 b | 6.2 ± 0.06 a | 2.5 ± 0.05 d | 19.6 ± 0.01 c | 27.7 ± 0.18 f |
| ***Ulva compressa* (G)** | AT | 1.6 ± 0.00 c | 32.7 ± 0.04 b | 5.5 ± 0.05 a | 2.4 ± 0.03 d | 17.7 ± 0.02 c | 28.7 ± 0.14 f |
| ***Ulva compressa* (G)** | JA | 1.8 ± 0.00 c | 31.6 ± 0.03 b | 6.2 ± 0.07 a | 2.3 ± 0.04 d | 18.6 ± 0.03 c | 29.7 ± 0.18 f |

* Elemental Ratio. ** Different litters near values of the same line express significant differences using the one-way ANOVA Tukey

HSD test ($p < 0.05$). *** B means brown seaweed, R means red seaweed, and G for green seaweed. **** RH means Rosh Hanikra, AT means Atlit, JA means Jisir Azarqah
